# Anti-metastatic potential of somatostatin analog SOM230: Indirect pharmacological targeting of pancreatic cancer-associated fibroblasts

**DOI:** 10.18632/oncotarget.9296

**Published:** 2016-05-12

**Authors:** Siham Moatassim-Billah, Camille Duluc, Rémi Samain, Christine Jean, Aurélie Perraud, Emilie Decaup, Stéphanie Cassant-Sourdy, Youssef Bakri, Janick Selves, Herbert Schmid, Yvan Martineau, Muriel Mathonnet, Stéphane Pyronnet, Corinne Bousquet

**Affiliations:** ^1^ Cancer Research Center of Toulouse (CRCT), INSERM UMR 1037-University Toulouse III Paul Sabatier, Toulouse, France; ^2^ Biochemistry-Immunology Laboratory, Faculty of Sciences Rabat, University Mohammed V - Agdal, Rabat, Morocco; ^3^ EA 3842 Laboratory, Medicine and Pharmacy Faculties, Limoges University, Limoges, France; ^4^ Pathology Department, Institut Universitaire du Cancer de Toulouse, Toulouse, France; ^5^ Oncology Global Development, Novartis Pharmaceuticals, Basel, Switzerland

**Keywords:** pancreatic cancer, tumor microenvironment, cancer-associated fibroblasts, metastasis, somatostatin analog SOM230

## Abstract

Pancreatic ductal adenocarcinoma (PDA) shows a rich stroma where cancer-associated fibroblasts (CAFs) represent the major cell type. CAFs are master secretors of proteins with pro-tumor features. CAF targeting remains a promising challenge for PDA, a devastating disease where treatments focusing on cancer cells have failed. We previously introduced a novel pharmacological CAF-targeting approach using the somatostatin analog SOM230 (pasireotide) that inhibits protein synthesis in CAFs, and subsequent chemoprotective features of CAF secretome. Using primary cultures of CAF isolated from human PDA resections, we here report that CAF secretome stimulates *in vitro* cancer cell survival, migration and invasive features, that are abolished when CAFs are treated with SOM230. Mechanistically, SOM230 inhibitory effect on CAFs depends on the somatostatin receptor subtype sst1 expressed in CAFs but not in non-activated pancreatic fibroblasts, and on protein synthesis shutdown through eiF4E-Binding Protein-1 (4E-BP1) expression decrease. We identify interleukin-6 as a SOM230-inhibited CAF-secreted effector, which stimulates cancer cell features through phosphoinositide 3-kinase activation. *In vivo*, mice orthotopically co-xenografted with the human pancreatic cancer MiaPaCa-2 cells and CAFs develop pancreatic tumors, on which SOM230 treatment does not inhibit growth but abrogates metastasis. Consistently, CAF secretome stimulates epithelial-to-mesenchymal transition in cancer cells, which is reversed upon CAF treatment with SOM230. Our results highlight a novel promising anti-metastatic potential for SOM230 indirectly targeting pancreatic cancer cell invasion through pharmacological inhibition of stromal CAFs.

## INTRODUCTION

Although tumorigenesis has classically been viewed as a largely cell-autonomous process involving genetically transformed cancer cells, the importance of stromal cell types populating the neoplastic microenvironment is now well accepted and needs to be taken into account for future therapeutic strategies [1, 2]. A critical stromal component for tumorigenesis is cancer-associated fibroblasts (CAFs). CAFs are phenotypically and functionally distinguishable from their normal counterparts in their differential expression and secretion of extracellular matrix (ECM) components and growth factors [3]. Several studies have demonstrated that normal fibroblasts have a role in maintaining epithelial homeostasis by suppressing proliferation and oncogenic potential of adjacent epithelia [4, 5]. However, following neoplastic transformation of epithelia, CAFs have been shown to promote tumor growth by inducing angiogenesis, inflammation, recruiting bone marrow–derived immunosuppressive cells, and remodeling the ECM [6, 7]. Interestingly, CAFs can even contribute to the resistance to antiangiogenic or chemotherapeutic therapy [8–10]. In cancers that present an extensive stroma reaction (also called desmoplasia), including pancreatic, breast, prostate or skin cancer, CAFs represent the major stromal cell population. By secreting pro-inflammatory factors as soon as the initiating hyperplastic phase of neoplasia, CAFs dialogue with epithelial cells to promote tumorigenesis [11]. This suggests that the therapeutic targeting of this cell population represents a promising strategy.

Somatostatin is a ubiquitous neuropeptide that inhibits many biological functions—including endocrine and exocrine secretion, gastric and intestinal motility, gallbladder contraction, angiogenesis, and cell proliferation—and induces apoptotic cell death. Its effects are mediated through five receptors that belong to the G-protein–coupled receptor family, sst1 to sst5 receptors, which bind natural somatostatin with high affinity. The short plasma half-life of natural somatostatin (~1.5 minutes) led to the development of analogs with higher stability, such as octreotide (Sandostatin; Novartis) (half-life of ~2 hours), that are used to treat pituitary and neuroendocrine tumors (NET). These analogs have high affinity for sst2 and sst5 receptors, lower affinity for sst3 receptor, and do not bind to sst1 or sst4 receptors. Pasireotide (SOM230) is a new, more stable analog with a plasma half-life of 12 hours [12]. It is considered a universal somatostatin analog because it binds to sst1, sst2, sst3, and sst5 with high affinity. Gastroenteropancreatic neuroendocrine tumors express high number of somatostatin receptors, which make somatostatin analogs ideal for diagnostic and therapeutic purposes. By contrast, our team has demonstrated the absence of expression of somatostatin receptors in pancreatic cancer cells [13]. Sst2 expression is lost during pancreatic acinar cell transformation upon acquisition of Kras oncogenic mutation [14], which may explain the inefficacy of somatostatin analogs that target sst2 to inhibit their survival [13].

Beside its inhibitory effects on tumor cells, somatostatin could affect tumor progression by impacting on the stromal compartment. Interestingly, sst1 and sst2 are expressed in pancreatic and liver fibroblasts during pancreatitis and hepatitis, where they have been described to transduce somatostatin inhibitory action on inflammation and fibrosis, by inhibiting the secretion of pro-inflammatory cytokines and the production of extracellular matrix [15, 16]. Our working hypothesis was that such fibro-inhibitory role of somatostatin may alter the pro-tumor activity of CAFs present in cancers where a desmoplasic reaction is prominent, including pancreatic cancer. Our results show that the pro-tumor effects of CAFs can be pharmacologically targeted using the SOM230 analog which presents a high affinity for sst1 that we previously found expressed in CAFs [17]. Strikingly, SOM230 demonstrates a potent anti-metastatic activity *in vivo* in an orthotpoic co-xenografted (pancreatic cancer cells + CAFs) mouse model of desmoplasic pancreatic cancer.

## RESULTS

### High protein synthesis in CAFs is responsible for the secretion of growth-promoting factors – phenotype reversion with the somatostatin analog SOM230

**Figure 1:**
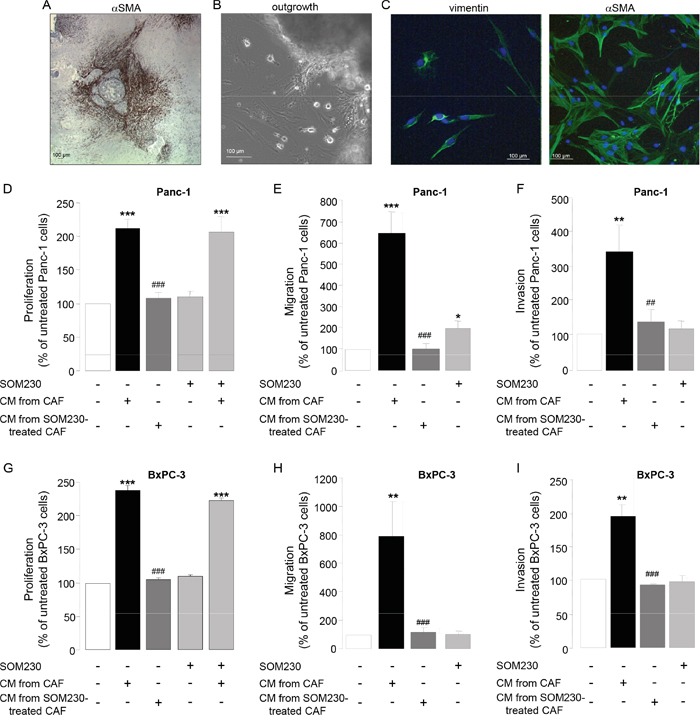
**A.** Immunohistochemistry using anti-αSMA antibody on a paraffin-embedded human pancreatic tumor section. **B.** CAF isolation by outgrowth from a human pancreatic tumor resection and *in vitro* primo-culture. **C.** Isolated CAF characterization *in vitro* by immunofluorescence using anti-vimentin (left panel) or -αSMA (right panel) antibody. **D-I.** CAFs were treated or not with SOM230 (10^−7^M) for 48 h. Conditioned media (CM) were collected. Pancreatic cancer cells (Panc-1, **D-F**, or BxPC-3, **G-I.** were incubated with the indicated CM, and cell viability was assessed by MTT **D, G.**, and cell migration **E, H.** and invasion **F, I.** using modified Boyden chambers. Results are presented for each treatment as a percentage of untreated cells (=100%) (n=3). *: effect of treatment *vs.* untreated cells; #: effect of incubation with CM from CAF *vs.* with CM from SOM230-treated CAFs.

PDA stroma is rich in CAFs that reside both inside the tumor and at the boundaries between the invasive cancer and the host pancreatic tissue. Secretion of growth factors and cytokines / chemokines by CAFs is critical for their pro-tumor functions. However underlying signaling mechanisms are unknown. To address this question, primary cultures of CAFs have been obtained from fifteen fresh human surgery-resected PDA tumors, of different stages and origin (Table 1), and that are positive for αSMA (Figure 1A). CAFs have been isolated by the outgrowth method: After a few days, cells migrate out of the tumor tissue piece by outgrowth (Figure 1B). Isolated cells show a fibroblast-like phenotype as confirmed by the expression of vimentin, and have an “activated” phenotype since nearly 100 % of them also express the marker αSMA (Figure 1C). Such phenotype is maintained throughout the passage of our primary cultures, until passage-10 where senescence occurs. Consistent with the literature, conditioned media (CM) isolated from those CAF primary cultures stimulate survival, migration and invasion of pancreatic cancer cells including Panc-1 and BxPC-3 (Figure 1D-1I), confirming their pro-tumor properties. By contrast, immortalization of a CAF primary culture through overexpression of hTERT reverted its growth-promoting effect on cancer cells, in correlation with the loss of expression of αSMA (Supplementary Figure 1A and 1B), confirming the StellaTUM recommendation to work with primary cultures of CAF only [18].

**Table 1:** List of PDAC human tumors, with the corresponding histopathological grading, used for CAF isolation

Tumors	Degree of differentiation of cancer cells	Stage
T1	well-differenciated	IIB
T2	moderately differenciated	IIB
T3	moderately differenciated	III
T4	moderately differenciated	III
T5	well-differenciated	IIA
T6	well-differenciated	IB
T7	poorly differentiated	IIB
T8	moderately differenciated	III
T9	poorly differentiated	III
T10	well-differenciated	III
T11	well-differenciated	III
T12	moderately differenciated	IIB
T13	well-differenciated	IB
T14	moderately differenciated	IIB
T15	well-differenciated	IB

Importantly, we previously observed in our primary culture of CAFs a high intrinsic activation of the PI3K-mTORC1 pathway, as revealed by the phosphorylation of Akt, S6, and of the translation inhibitor 4E-BP1, resulting in high protein synthesis rates [17]. We have therefore questioned whether impacting on this pathway in CAFs may attenuate their pro-tumor features by affecting the synthesis and secretion of growth factors. We have previously demonstrated that somatostatin, a natural peptide physiologically secreted by δ-pancreatic islet cells is able to inhibit the PI3K-Akt pathway in different cells where this pathway is activated [19–22]. Strikingly, CAF treatment with a novel pan-somatostatin analog SOM230 (Pasireotide^®^ Novartis) does not directly affect their survival (Supplementary Figure 2), nor the survival of pancreatic cancer cells whether or not they are also exposed to CAF CM (Figure 1D, 1G, bars 4 & 5). However, when CAF are previously treated with SOM230, their CM fail to stimulate Panc-1 and BxPC-3 cell survival, migration and invasion (Figure 1D-1I, bar 3). These results demonstrate that CAF treatment with SOM230 reverses the pro-tumoral features of their CM. SOM230 inhibitory effect on cancer cells is indirect, acting solely on CAFs by regulating the composition in soluble growth-promoting factors of their secretome.

**Figure 2:**
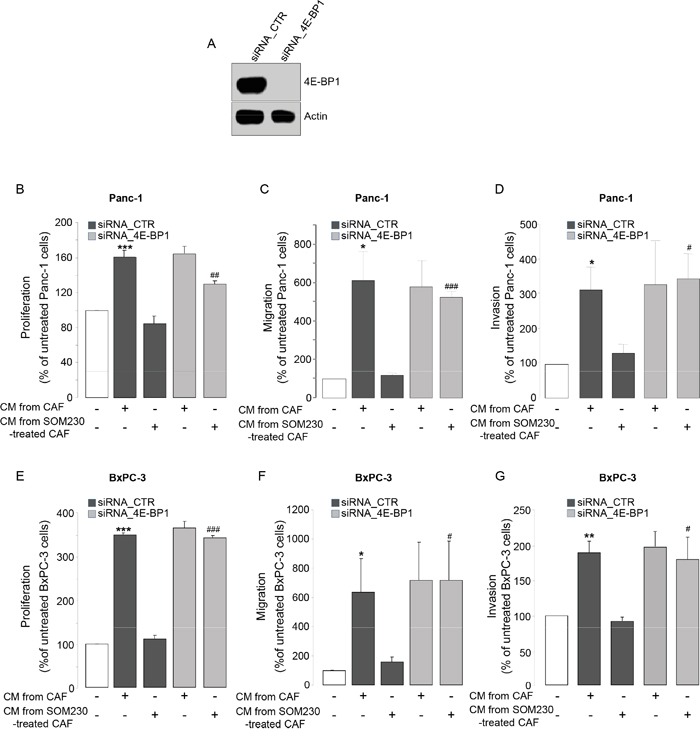
**A.** Immunoblotting using an anti-4E-BP1 or -actin (loading control) antibody of protein extracts from siCTR or si4E-BP1-transfected CAFs (representative of n=3). **B-G.** Panc-1 **B-D.** and BxPC-3 **E-G.** cell viability **B, E.** was assessed by MTT, and cell migration **C, F.** or invasion **D, G.** using modified Boyden chambers. Cells were incubated in the presence of CM from untreated or SOM230-treated CAFs transfected with the siRNA_CTR or siRNA_4E-BP-1. Results are presented as a percentage of the survival, migration or invasion observed in the condition where cancer cells were not exposed to CAF CM (=100%) (n=3). *: effect of treatment *vs.* untreated cells; #: siRNA CTR *vs.* siRNA 4E-BP1.

We then addressed how SOM230 regulates the expression of soluble growth factors in CAF CM, hypothesizing that it may rely on SOM230 inhibitory action on protein synthesis, as we previously reported [17]. We abrogated the expression of the inhibitor of mRNA translation 4E-BP1 by RNA interference (Figure 2A). Promoting effects of CAF CM on survival, migration and invasion of Panc-1 and BxPC-3 cells are maintained upon 4E-BP1 expression knock-down (Figure 2B-2G, compare bars 2 & 4). SOM230 inhibitory effect on these CAF features is not affected in the siRNA-CTR condition, but is reversed upon 4E-BP1 invalidation (Figure 2B-2G, compare bars 3 & 5). These results demonstrate that inhibition by SOM230 of the pro-tumor features of CAF CM is 4E-BP1-dependent.

### SOM230 reverses the pro-tumor properties of CAF secretions by activating the somatostatin receptor sst1

**Figure 3:**
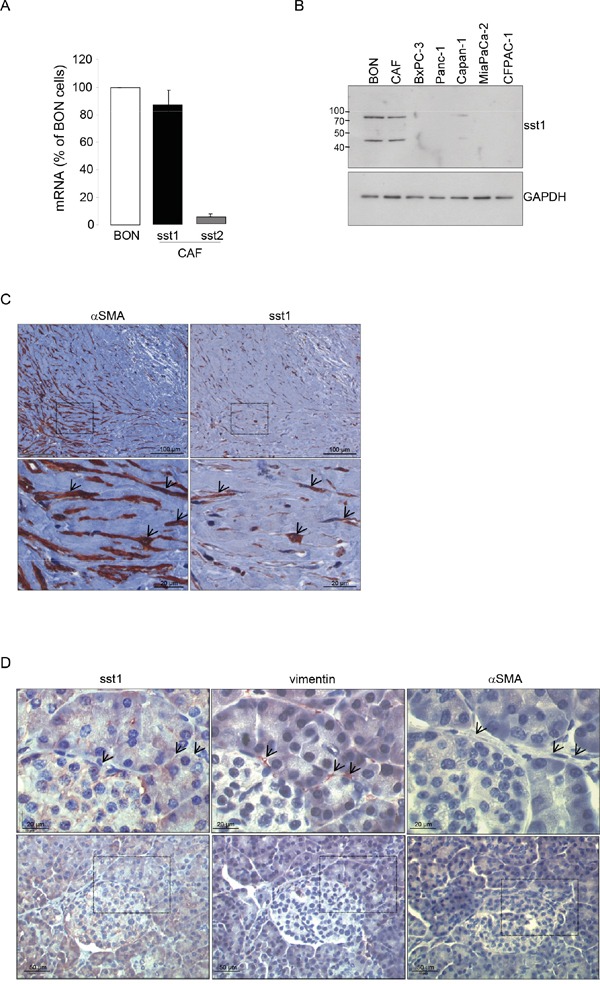
**A.** Expression of the somatostatin receptor subtype 1 (sst1) and subtype 2 (sst2) analyzed by RT-qPCR in the human pancreatic endocrine cell line BON and in CAFs isolated from 10 different patients. **B.** Immunoblotting of protein extracts from BON, CAFs, and human pancreatic cancer cell lines (BxPC-3, Panc-1, Capan-1, MiaPaCa-2, CFPAC-1) using anti-sst1 or GAPDH (loading control) antibody (representative of n=3). **C.** Immunohistochemistry analyses using an anti-αSMA or -sst1 antibody in paraffin-embedded pancreatic cancer serial sections (one representative field of 10 different PDA samples). **D.** Immunohistochemistry using an anti-sst1, vimentin, or αSMA antibody in serial sections of paraffin-embedded normal human pancreas (one representative field of 10 different normal pancreas, adjacent to tumors). Arrows point to vimentin-positive but αSMA-negative fibroblasts also presenting no stain for sst1.

**Figure 4:**
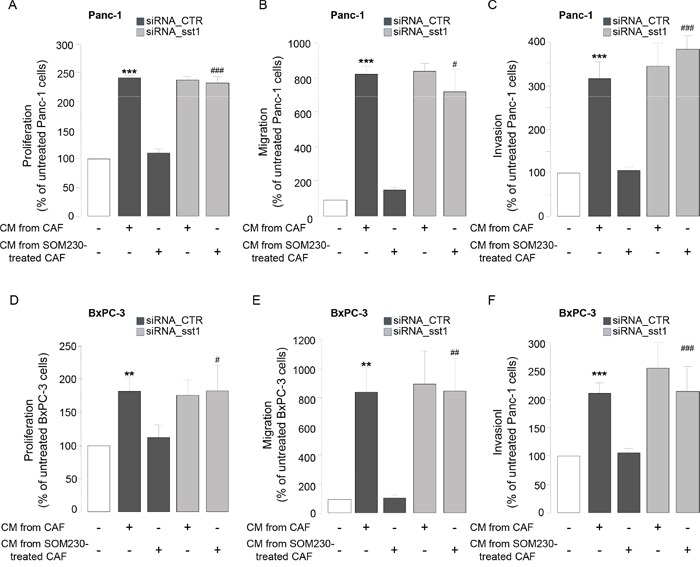
**A-F.** Panc-1 **A-C.** and BxPC-3 **D-F.** cell viability **A, D.** was assessed by MTT, and cell migration **B, E.** or invasion **C, F.** using modified Boyden chambers. Cells were incubated in the presence of CM from untreated or SOM230-treated CAFs transfected with the siRNA_CTR or siRNA_sst1. Results are presented as a percentage of the survival, migration or invasion observed in the condition where cancer cells were not exposed to CAF CM (=100%) (n=3). *: effect of treatment *vs.* untreated cells; #: siRNA CTR *vs.* siRNA sst1.

To address how SOM230 affects CAF features, we investigated expression levels of somatostatin family receptors (sst1 to sst5) in CAFs. Quantitative RT-PCR analyses (Figure 3A), confirmed by Western-blot (Figure 3B), indicate that sst1 is expressed in isolated CAFs from PDA, but not in human pancreatic cancer cell lines, and in a level similar as in human neuroendocrine pancreatic tumor BON cells known to express high levels of sst1 and sst2 [23]. In contrast, sst2 is poorly expressed, and sst3, sst4 and sst5 are not expressed (Figure 3A & not shown). Immunohistochemistry analysis on serial PDA sections (n=10) confirm *in situ* that the same stromal cells positive for αSMA are also expressing sst1 (Figure 3C, arrows). In contrast, vimentin-positive fibroblasts present in healthy pancreas are αSMA-negative and do not also express sst1 (Figure 3D, arrows). We then investigated whether sst1 transduces the inhibitory effect of SOM230 on the pro-tumor properties of CAF secretome. CM obtained from CAFs treated with octreotide, another somatostatin analog presenting a high binding-affinity for sst2 but unable to activate sst1, keep their stimulatory effect on pancreatic cancer cell survival (Supplementary Figure 3A and 3B), suggesting that sst1 but not sst2 mediates SOM230 action. To fully address sst1 function in CAFs, sst1 expression has been knock-down using RNA interference. Among the three siRNA targeting sst1, only siRNA-sst1a abrogates sst1 expression, as shown by western blot and immunohistochemistry (Supplementary Figure 4A and 4B). Consistently, siRNA-sst1a, but not siRNA-sst1b, reverses SOM230 inhibitory effects on the growth-promoting activities of CAF secretome (Supplementary Figure 4C), confirming specificity of siRNA and antibodies. One of the three siRNA targeting sst1 (siRNA_sst1a) that fully abrogates sst1 expression has been used for further functional studies on pancreatic cancer cells. As expected, CM obtained from CAFs transfected with a control siRNA (siRNA_CTR) stimulate survival, migration and invasion of pancreatic Panc-1 and BxPC-3 cells, whereas CAF treatment with SOM230 reverses the pro-tumor features of their secretome (Figure 4A-4F, bars 1-3). By contrast, upon sst1 expression knockdown, CAFs are refractory to this inhibitory effect of SOM230, since their secretions keep their properties to stimulate pancreatic cancer cell survival, migration and invasion (Figure 4A-4F, compare 3 & 5). Sst1 expression knockdown does not affect basal pro-tumor effect of CAF secretome (Figure 4A-4F, compare bars 2 & 4). These results demonstrate that SOM230 inhibitory effect on CAF secretome is entirely dependent on sst1.

### IL-6 is a critical soluble factor responsible for the pro-tumor activities of CAF secretions which is druggable by SOM230

**Figure 5:**
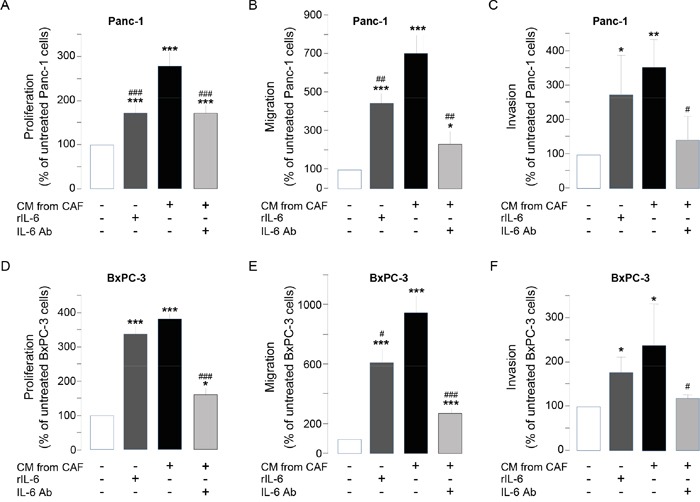
**A-F.** Panc-1 **A-C.** and BxPC-3 **D-F.** cell viability **A, D.** was assessed by MTT, and cell migration **B, E.** or invasion **C, F.** using modified Boyden chambers. Cells were incubated in the presence of recombinant IL-6 (rIL-6), or CM from untreated or SOM230-treated CAFs in the presence or not of the IL-6 neutralizing antibody (IL-6 Ab). Results are presented as a percentage of the survival, migration or invasion observed in the condition where cancer cells were not exposed to rIL-6 or CAF CM (=100%) (n=3). *: effect of treatment *vs.* untreated cells; #: effect of IL-6 Ab.

We have already identified interleukin-6 as a soluble factor highly secreted by CAFs (> 1 ng secreted per 10^6^ CAFs, Supplementary Figure 5A), but not by pancreatic cancer cells, and whose translation / secretion is inhibited by CAF treatment with SOM230 [17]. Sst1 role on SOM230-mediated inhibition of IL-6 secretion by CAFs was here shown in siRNA_sst1a-transfected CAFs which demonstrate partial rescue of IL-6 production upon SOM230 treatment, whereas, as expected, IL-6 secretion was not affected by sst1 knockdown in untreated CAFs (Supplementary Figure 5B). The importance and functionality of IL-6 secreted by CAFs was tested on pancreatic cancer cell survival, migration and invasion: 1 ng of recombinant IL-6 (rIL-6) stimulated the survival, migration and invasion of Panc-1 and BxPC-3 cells, although less efficiently than CAF secretions. Conversely, blocking IL-6 activity in CAF secretions partially but significantly reversed the stimulatory effects of CAF secretions (Figure 5A-5F). Taken together, these results demonstrate the role of IL-6 as a critical CAF-secreted factor responsible for CAF pro-tumor actions on pancreatic cancer cells.

### Activation of the PI3K pathway in pancreatic cancer cells is important for CAF pro-tumor effects

**Figure 6:**
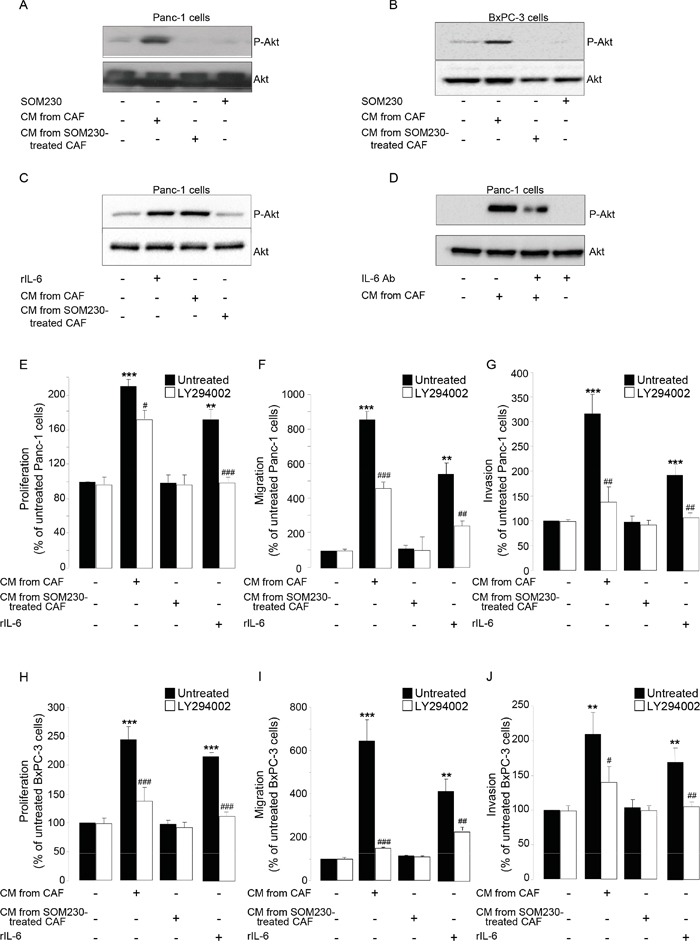
**A-D.** Immunoblotting using an anti-P-Akt, or Akt (loading control) antibody of protein extracts from Panc-1 cells **A, C-D.** or BxPC-3 cells **B.** treated or not for 15 min with SOM230 (10^−7^M), or with recombinant IL-6 (rIL-6), or with IL-6 Ab, or with CM from untreated or SOM230-treated CAFs in the presence or not of the IL-6 neutralizing antibody (IL-6 Ab) (representative of n=3). **E-J.** Panc-1 **E-G.** and BxPC-3 **H-J.** cell viability **E, H.** was assessed by MTT, and cell migration **F, I.** or invasion **G, J.** using modified Boyden chambers. Cells were pre-treated or not for 30 min with LY294002, and then incubated in the presence of recombinant IL-6 (rIL-6), or CM from untreated or SOM230-treated CAFs. Results are presented as a percentage of the survival, migration or invasion observed in the condition where cancer cells were not exposed to rIL-6 or CAF CM (=100%) (n=3). *: effect of treatment *vs.* untreated cells; #: effect of LY294002.

We then investigated which signaling pathway(s) is (are) stimulated in pancreatic cancer cells upon exposure to CAF secretions, but indirectly inhibited by SOM230 through its action on CAFs. Our results indicated that one major signaling pathway potently induced by CAF secretions in pancreatic cancer cells is the PI3K-Akt pathway, as demonstrated using phosphorylation of Akt as a readout (Figure 6A-6B); Akt phosphorylation is also observed upon treatment of pancreatic cancer cells with rIL-6 (Figure 6C), but not with the secretions of CAFs previously treated with SOM230 (Figure 6A-6C). SOM230 has no effect by itself on Akt phosphorylation in pancreatic cancer cells (Figure 6A-6B). Blocking IL-6 activity (using a blocking antibody) in CAF secretions partially reversed phosphorylation of Akt stimulated with CAF secretions (Figure 6D), consistent with an activation at least dependent on IL-6. Inhibition of PI3K activity using LY294002, a specific PI3K inhibitor, potently inhibited pancreatic cancer cell survival, migration and invasion induced by CAF secretions or by rIL-6 (Figure 6E-6J), demonstrating the importance of PI3K activation in the pro-tumor effects of CAF secretions.

### SOM230 is antimetastatic *in vivo*

**Figure 7:**
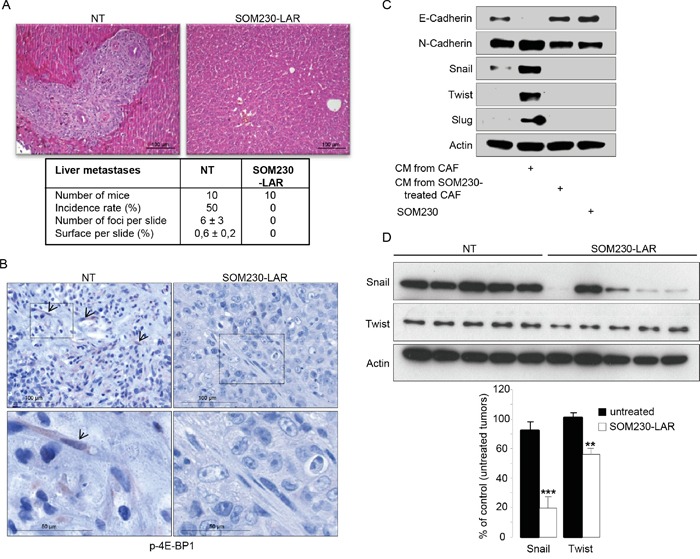
**A, B.** Mice orthotopically co-xenografted with pancreatic cancer cells and CAFs, and treated or not with SOM230-LAR. **A.** Hemalun-eosin coloration of the liver: On the left panel (untreated condition, NT) a liver metastasis is visible. Below, a table indicating, per treatment group, the number of mice, the metastasis incidence rates (%), the number of metastasis foci per slide, and the metastasis surface per slide (%). **B.** Immunohistochemistry analyses using an anti-p-4E-BP1 antibody in paraffin-embedded pancreatic tumor serial sections. (one representative field of 10 different tumors). **C.** Immunoblotting using an anti-E-cadherin, or N-cadherin, or Snail, or Twist, or Slug, or actin (loading control) antibody of protein extracts from MiaPaCa-2 cells treated or not with SOM230 (10^−7^M), or with CM from untreated or SOM230-treated CAFs (representative of n=3). **D.** Immunoblotting using an anti-Snail or Twist, or actin (loading control) antibody of protein extracts from MiaPaCa-2 cell + CAF xenografted tumors in five mice treated or not with SOM230-LAR (upper panel), and quantification of the bands using the Image J software (lower panel).

To ascertain our proof of concept that SOM230 is able to inhibit the pro-tumor properties of CAFs, SOM230 efficacy has been tested *in vivo*, in athymic mice that have been subcutaneously or orthopically (pancreas) xenografted with the human pancreatic cancer MiaPaCa-2 cells together with CAFs. Mice have been treated or not every 28 days with 80 mg/kg of SOM230-LAR, a long acting releasing form of SOM230. We previously showed in both models that the exponential growth of combined cancer cells + CAFs tumors was not slowed down in mice treated with SOM230, as compared to untreated mice [17]. In contrast, we here show that liver metastases observed in 50 % of mice orthotopically co-xenografted with cancer cells and CAFs after 3 weeks of engraftment (Figure 7A, Table 1) were not observed in any of the mice treated with SOM230. As surrogate *in vivo* marker in CAFs of the SOM230 inhibitory effect on protein synthesis, we observed a decreased phosphorylation of 4E-BP1 in the SOM230-treated, but not untreated, tumors (Figure 7B). We then hypothesized that SOM230, by affecting protein synthesis in CAFs, and subsequently the composition of their CM (including IL-6 concentration), indirectly inhibits epithelial-to-mesenchymal transition (EMT) in cancer cells, a critical process for cancer cell migration and invasion [24]. Expression of EMT markers in pancreatic cancer cells *in vitro* incubated with CM from CAFs treated or not with SOM230 was checked. Whereas expression in cancer cells of N-cadherin, Twist, Slug and Snail was upregulated, and expression of E-cadherin was downregulated, by CAF CM, CM from SOM230-treated CAFs was inefficient to promote EMT (Figure 7C). SOM230 did not have any direct effect by itself on cancer cell EMT markers. Our data were confirmed *in vivo* where SOM230-treated tumors demonstrated drastic decreased in Snail expression, and modest for Twist, as compared to untreated-tumors (Figure 7D). Altogether, these results demonstrate the efficacy of SOM230 to target CAF *in vivo* and prove its putative therapeutic interest to slow-down CAF-stimulated pro-invasive features.

## DISCUSSION

As CAFs are closely related to cancer survival, metastasis or drug resistance features, these cells represent a promising therapeutic target. Our present data provide a pharmacological strategy for targeting CAF prometastatic features with SOM230, the only clinically approved (European Union and United States) somatostatin analog that activates the somatostatin receptor sst1 with high affinity (IC50 = 9.3 nmol / L). We recently published that SOM230 represents a novel anti-stromal targeted therapy with promising potential in chemosensitization for PDA patients. Indeed, we demonstrated that, by activating the sst1 receptor expressed in CAFs, SOM230 inhibits protein synthesis thereby decreasing the concentration of CAF secreted factors with chemoprotective features for pancreatic cancer cells [17]. Analyses on serial sections of human PDA and healthy pancreatic tissue here confirms that αSMA-expressing cells also express sst1 in PDA, and shows that the vimentin-positive but αSMA-negative cells, which represent the population of pancreatic stellate cells (PSCs, quiescent cells) in healthy pancreas, do not express sst1. Mechanisms for sst1 expression upregulation during PSC activation into CAFs is currently under investigation. It was reported that HSCs (hepatic stellate cells) express sst1, and also the sst2 and sst3 receptor subtypes, but only when they are activated into myofibroblasts (not in their quiescent state) [16]. Immunohistochemical staining of PDA tissues show that CAFs do not express other somatostatin receptors (not shown), in accordance with the *in vitro* RT-qPCR results detecting only very small amount of sst2 mRNA (as compared to level observed in the human BON neuroendocrine cell line known to express sst2) [25]. This may explain the failure of sst2-selective somatostatin analogs such as octreotide to treat and detect PDA (octreoscan) in patients, since sst2 is not expressed in the two PDA major cell types, i.e. pancreatic cancer cells and CAFs [14, 19]. Consistently, in contrast to SOM230, octreotide is inefficient to inhibit CAF pro-tumor features. Sst1 expression knockdown in CAFs abrogates SOM230 inhibitory effect on CAF pro-tumor features, confirming the critical role of sst1, the only somatostatin receptor expressed in these cells, to transduce SOM230 action.

We here show that SOM230 has no effect on CAF proliferation or survival. This result may seem surprising given the known inhibitory effects of somatostatin analogs on cell growth [13], but could be explained by the very low proliferation rate of these cells [17]. On another hand, it appears that SOM230 negatively regulates the pro-tumor features of CAF secretions, i.e. their ability to stimulate the proliferation, migration and invasion of pancreatic cancer cells. Although CAFs are known mainly for their high capacity of protein secretion, nothing was known on protein synthesis and its regulation in these cells. We recently demonstrated that the protein synthesis mTOR/4E-BP1 regulatory pathway is highly activated in CAFs isolated from human PDA resections, resulting in elevated synthesis of secreted proteins including IL-6. By inhibiting the mTOR/4E-BP1 pathway, SOM230 abolished chemoresistance induced by CAF secretome [17]. Similarly, we here demonstrate that SOM230 inhibitory effect on the pro-tumor features of CAF secretions depends on 4E-BP1 regulation. Indeed, 4E-BP1 expression knockdown abrogates those SOM230 inhibitory benefits. Interestingly, strategy aimed at targeting 4E-BP1 in pancreatic cancer cells is doomed to failure according to 4E-BP1 loss of expression in these cells [26]. Our results suggest that such a therapeutic strategy might be promising in CAFs. An analysis of factors differentially secreted by CAFs treated or not with SOM230 revealed that IL-6 is one major factor whose mRNA translation is dramatically inhibited by SOM230 [17]. Mechanisms previously known for IL-6 expression regulation in CAFs include transcriptional activation of IL-6 promoter through a sonic hedgehog – GLI1 pathway [27]. Negative regulation by SOM230 of IL-6 protein synthesis (and subsequent secretion) by CAFs is translational and depends on the inhibition of the mTOR/4E-BP1 pathway [17]. In addition to its role in the regulation of inflammation and immune response in PDA [27, 28], we here show that IL-6 has a significant stimulatory effect on the proliferation, migration and invasion of pancreatic cancer cells. Moreover, neutralization of IL-6 partially reverses CAF secretome's pro-proliferative, pro-migratory and pro-invasive properties on cancer cells. Our results are consistent with the recent data demonstrating that IL-6 is required for pancreatic cancer progression and emerges as a potential therapeutic target [28, 29]. Interestingly, we previously demonstrated that *in vivo* PDAC mouse model treatment with SOM230 decreases IL-6 production by CAFs and IL-6 plasma concentrations [17].

Several signaling pathways in pancreatic cancer cells may be affected by CAF secretome, including canonical pathways leading to cell proliferation / survival / migration and invasion. Our investigation was oriented towards the PI3K pathway, which is highly active in 50% of cases of PDA despite the absence of obvious mutations in this pathway detected in PDA patient tumors [30]. The PI3K pathway is critically involved in pancreatic carcinogenesis as recently demonstrated using genetically-engineered mouse models [31]. Intracrine signaling loops downstream of oncogene activation and tumor suppressor inactivation sustains intrinsic PI3K activation in pancreatic cancer cells [14, 31]. Paracrine loops involving the microenvironment may be involved. Our results here show that CAF secretome activates the PI3K pathway, which plays a key role in the response of pancreatic cancer cells to pro-proliferative, pro-migratory and pro-invasive of CAF-derived stimuli including IL-6.

Sub-cutaneous or intra-pancreatic co-xenografting of human pancreatic cancer cells together with CAFs in athymic mice followed by mouse treatment with SOM230 show that SOM230 does not control tumor growth. Nevertheless, liver metastasis which occurs in 50 % of mice orthotopically engrafted with MiaPaCa-2 cells is abolished upon SOM230 treatment. No metastases (liver or lung) are observed in the subcutaneous engrafted model. Expression of EMT proteins is upregulated in pancreatic cancer cells upon *in vitro* exposure to CAF secretome. Conversely, expression of those EMT proteins is not induced in cancer cells exposed to SOM230-treated CAF secretome, providing a putative mechanism for SOM230 antimetastatic potential.

SOM230, also known as Pasireotide or Signifor^®^, is of particular interest since it has already received a marketing authorization (for Cushing's disease [32]). SOM230 also presents the advantage to specifically target activated sst1-expressing, but not quiescent, fibroblasts enhancing its therapeutic interest. From these results and our recent published data [17], we conclude that pharmacological targeting of CAFs with SOM230 represents a promising antimetastatic and chemosensitization strategy for PDA patients.

## MATERIALS AND METHODS

### Human cancer-associated fibroblasts isolation and cell culture

Human pancreatic tumor tissues were obtained from the Pathology Department of Toulouse and Limoges Hospitals, France, from patients undergoing resections for pancreatic adenocarcinoma. This study was approved by the ethic committee of the Institutions. Patient samples were obtained after informed consent in accordance with the declaration of Helsinki and stored at the « CRB cancer des Hôpitaux de Toulouse » collection. According to the french law, CRB cancer collection has been declared to the ministry of higher education and research (DC 2009-989; DC-2011-1388) and obtained a transfer agreement (AC-2008-820; AC-2011-130) after approbation by ethical committees. Clinical and biological annotations of the samples have been declared to the CNIL (Comité National Informatique et Libertés). Pancreatic tumor tissue was collected in Dulbecco's Modified Eagle's medium F12 (DMEM/F12 Lonza) containing 20% Foetal Calf de-complemented serum (FCS, Life Technologies). The culture medium was changed after the cells had attached. Cells were isolated from these samples using the outgrowth method described by Bachem et al. [33]. Ductal pancreatic adenocarcinoma cell lines, Panc-1, BxPC-3 and MiaPaCa-2, are grown in RPMI and DMEM (4,5 g/L glucose, Lonza), respectively, containing 10% FCS. Cells were cultured at 37°C, 5% CO2 in a humid atmosphere. All media were supplemented with antibiotics (5 IU / mL penicillin and streptomycin, 2.5 mg / mL of fungizon and 2, 5 μL / mL plasmocin) and L-glutamine (2 mM) (Life Technologies). For *in vivo* subcutaneous or pancreatic xenografting, Panc-1 or MiaPaCa-2-GLuc cells expressing the secreted Gaussia Luciferase were used, respectively, as previously described [17].

### Reagents

Antibodies used are as follow: Anti-αSMA (Epitomics), anti-Vimentin, anti-IL-6 (Abcam), sst1 (generated in Novartis Pharma laboratory (Basel, Switzerland) [25, 34], anti-GAPDH (Santa Cruz), anti-β-Actin (Sigma-Aldrich), anti-p-Akt, anti-Akt, anti-4E-BP1 (Cell signaling). Drugs used are as follow: SOM230 (10^−7^M, Novartis Pharma, Basel, Switzerland); recombinant human IL-6 (1 ng/mL, PreproTech).

### Immunohistochemitry

Serial sections of formalin-fixed, paraffin-embedded human tumor samples were incubated with anti-sst1, anti-vimentin or anti-αSMA antibody. Reactions were amplified with ImmPRESS Peroxidase Polymer Detection Reagents (VECTOR Laboratories). A solution 3-amino-9-ethylcarbazole (AEC) or 3,3-Diaminobenzidine (DAB) were used as chromogen and sections were counterstained with hematoxylin. Images were captured using an Eclipse E400 microscope (Nikon) and Explora Nova Morpho Expert/Mosaic Software.

### CAF CM

CAF CM were made at a concentration of 10^6^ cells / 5 ml of DMEM/F12 (0% FCS) treated or not with 10^−7^M of SOM230. 48 hours later, CM were collected, centrifuged (2 000 rpm, 5 min) and filtered (0.2 μm) prior to incubation with cancer cells. When indicated, CM from untreated CAF were pre-treated for thirty min with 1 ng/mL of human IL-6 neutralizing IgA monoclonal antibody (Abcam) prior to incubation with cancer cells.

### MTT assay

Tumor cells were seeded in 96-well plates (20 000 cells/well). After adhesion, cells were washed and starved. The following day, cells were treated with gemcitabine and stimulated or not with CAF CM as indicated. MTT (3-(4,5-dimethylthiazol-2-yl)-2,5-diphenyltetrazolium bromide, Life Technologies) was added to each well at 0,5 mg/mL for 2 hours. Hundred μl of DMSO (dimethyl sulfoxide) were added for 1 h to each well. Viability was estimated by measuring absorbance at 570 nm on MRX plate reader (Dynex Technologies).

### Migration and invasion assays

Migration and invasion assays were performed using transwell inserts (8,0 μm pore size, BD Falcon), coated with matrigel (BD Biosciences) for invasion. Cells were seeded on the upper chamber. Chemoattractant was added (serum) to the lower chamber. Panc-1 and BxPC-3 cells were allowed to migrate for 20 h. Cells that migrated to the lower side of the membrane were fixed with 4% paraformaldehyde and stained with crystal violet (0.2 %) in 20 % methanol. Quantification of the membrane surface area covered by migrated / invaded cells was performed with Morpho Expert software, Explora Nova.

### Western blot

Cells were lysed in lysis buffer (20 mM Tris, pH 7.5, 150 mM NaCl, 1 mM EDTA, 1% NP40, 1 mM sodium orthovanadate, 1 mM NaF and a cocktail of protease inhibitors, Roche). Protein extract concentration was measured using Protein Assay reagent (Bio-Rad), and equal amount of proteins were resolved by SDS-PAGE and electroblotted onto nitrocellulose membranes. Membranes were blocked in 5% powdered milk in Tris-buffered saline with 0.1% Tween 20 (TBST) for 1 h followed by incubation with primary antibodies overnight at 4°C. Membranes were then washed with TBST three times and incubated with horseradish peroxidase-coupled secondary antibody for 1 h at room temperature, washed again, and treated with enhanced chemiluminescence prior to detectionon x-ray film.

### Real-time quantitative RT-PCR

Total RNA was extracted with RNeasy kit (Qiagen) and equal amount of RNAs were reverse-transcribed using Superscript One Step RT-PCR kit (Invitrogen^®^) and resulting cDNAs were used in qRT-PCR using SYBR green (Applied Biosystems) according to the manufacturer's instructions. Primer sequences (Invitrogen) were as follow: SSTR1 sense 5′-CAC ATT TCT CAT GGG CTT CCT-3′, reverse 5′-ACA AAC ACC ATC ACC ACC ATC 3′; SSTR2 sense 5′ –GGC ATG TTT GAC TTT GTG GTG-3′, reverse 5′-GTC TCA TTC AGC CGG GAT TT-3′; SSTR3 sense 5′-TGC CTT CTT TGG GCT CTA CTT-3′, reverse 5′-ATC CTC CTC CTC AGT CTT CTC C-3′; SSTR4 sense 5′-CGT GGT CGT CTT TGT GCT CT-3′, reverse 5′-AAG GAT CGG CGG AAG TTG T-3′; SSTR5 sense 5′-CTG GTG TTT GCG GGA TGT T-3′, reverse 5′-GAA GCT CTG GCG GAA GTT GT-3′; and IL6 sense 5′-CTG ACC CAA CCA CAA ATG CC-3′, reverse 5′-GGT TCT GTG CCT GCA GCT TC 3′. Target gene expression was normalized by using HPRT expression as an internal control.

### RNA interference

hCAFs were transfected with siRNA targeting (25 nM) SSTR1 (four individual siRNAa-d, Dharmacon.) or (50 nM) 4E-BP1 (Applied Biosystems, forward 50-CAAGAACGAACCCUUCCUU-3′ and reverse using respectively DharmaFECT1 reagent (Dharmacon) and siPort NeoFx transfection reagent (Applied Biosystems), respectively, according to the manufacturer's instructions.

### Tumor growth (co-xenografts in nude mice)

#### Pancreatic cancer cells and CAF co-xenografting

Pancreatic cancer cells and CAFs were trypsinized, washed and resuspended in sterile PBS. A 1:3 mix of pancreatic cancer cells (10^6^) and CAFs (3.10^6^) were subcutaneously (Panc-1 cells) or orthotopically (intra-pancreatic) (MiaPaCa-2-Gluc cells) injected in 100 μL or 50 μL PBS, respectively, of 4-week-old female nude mice (NMRI-nu; Janvier) (n=10 mice per condition) [35]. Mice were anesthetized by inhalation of isoflurane. Treatments started one week after grafting when mice have been randomized in the two group treatments (untreated and SOM230-LAR treated, n=10 mice per group), as previously described [17].

#### Treatments

Mice have been treated s.c. with SOM-LAR (80 mg/kg) once every 28-days.

Tumor volumes of subcutaneous tumors were calculated as 0,523 x l^2^ x L. All experiments were done in accordance with the principles and guidelines established by INSERM Anexplo UMS006 and were approved by the institutional and regional animal care and use committees.

#### Metastasis analyses

Formalin-fixed paraffin-embedded livers were cut in whole (4 μm -thick sections), and 1 slide out of 25 (every 100 μm) has been Hemalun-eosin-colored and examined for the presence and quantification of metastasis foci (mean number per section, and mean surface as a % of total liver surface), using the NanoZoomer Digital Pathology Virtual Slide Viewer (Hamamatsu).

### Statistical analyses

Statistical analyses were performed by comparing two by two independent conditions (with homogeneous variances) using an unpaired parametric T-test. All values are mean ± SEM. Differences were considered statistically significant when *P* < 0.05 (*, *P* < 0.05; **, *P* < 0.01; ***, *P* < 0.001).

## SUPPLEMENTARY FIGURES


