# Supplementary material for: Anti-metastatic potential of somatostatin analog SOM230: Indirect pharmacological targeting of pancreatic cancer-associated fibroblasts
Source: Oncotarget. 2016 May 12;7(27):41584–98. doi: 10.18632/oncotarget.9296 (PMC5173080; doi:10.18632/oncotarget.9296)
Supplement: Supplementary file 1 [file oncotarget-07-41584-s001.pdf]

Anti-metastatic potential of somatostatin analog SOM230: Indirect pharmacological targeting of pancreatic cancer-associated fibroblasts

SUPPLEMENTARY FIGURES

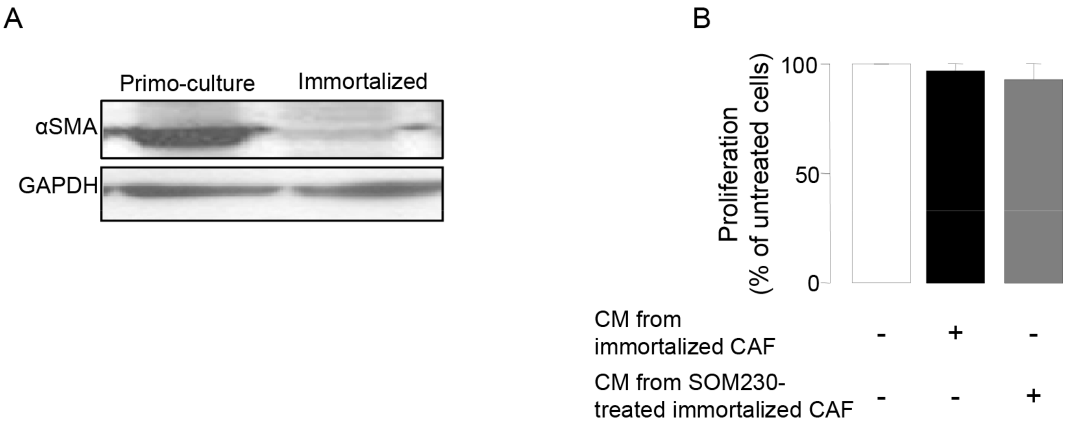

Supplementary Figure S1: CAFs immortalized through overexpression of hTERT. A. Immunoblotting using an anti-αSMA or GAPDH (loading control) antibody of protein extracts from a primary culture of CAFs or from immortalized CAFs. **B.** Panc-1 cell viability was assessed by MTT. Cells were incubated in the presence of CM from immortalized CAFs treated or not with SOM230. Results are presented as a percentage of the survival observed in the condition where cancer cells were not exposed to CAF CM (=100%) (n=3).

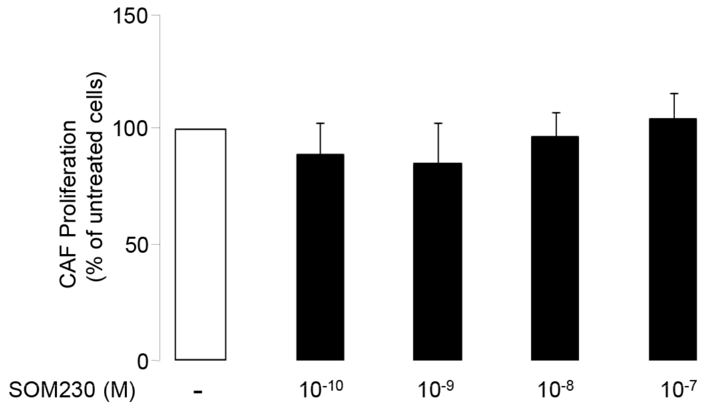

Supplementary Figure S2: CAF viability was assessed by MTT. Cells were treated or not for 48 h with SOM230 (10^{-10} - 10^{-7} M).

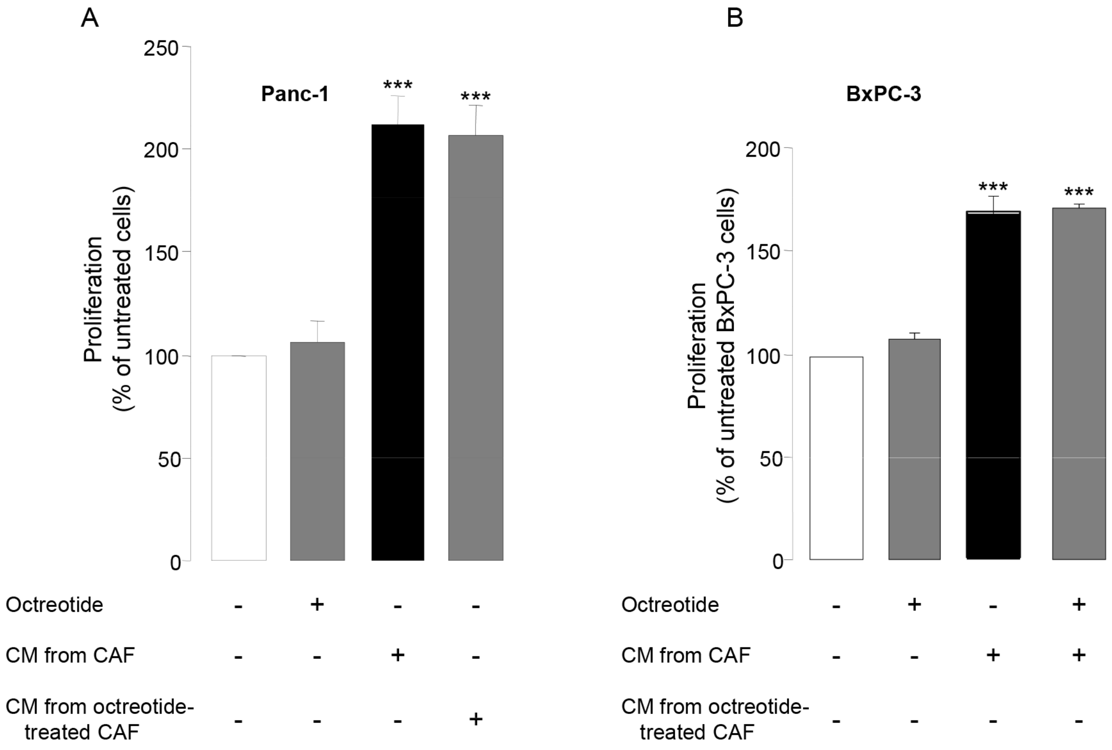

Supplementary Figure S3: CAFs were treated or not with octreotide (10^{-8}M) for 48 h. Conditioned media (CM) were collected. Pancreatic cancer cells (Panc-1, **A.**, or BxPC-3, **B.**) were incubated with the indicated CM, and cell viability was assessed by MTT. Results are presented for each treatment as a percentage of untreated cells (=100%) (n=3). *: effect of treatment *vs.* untreated cells.

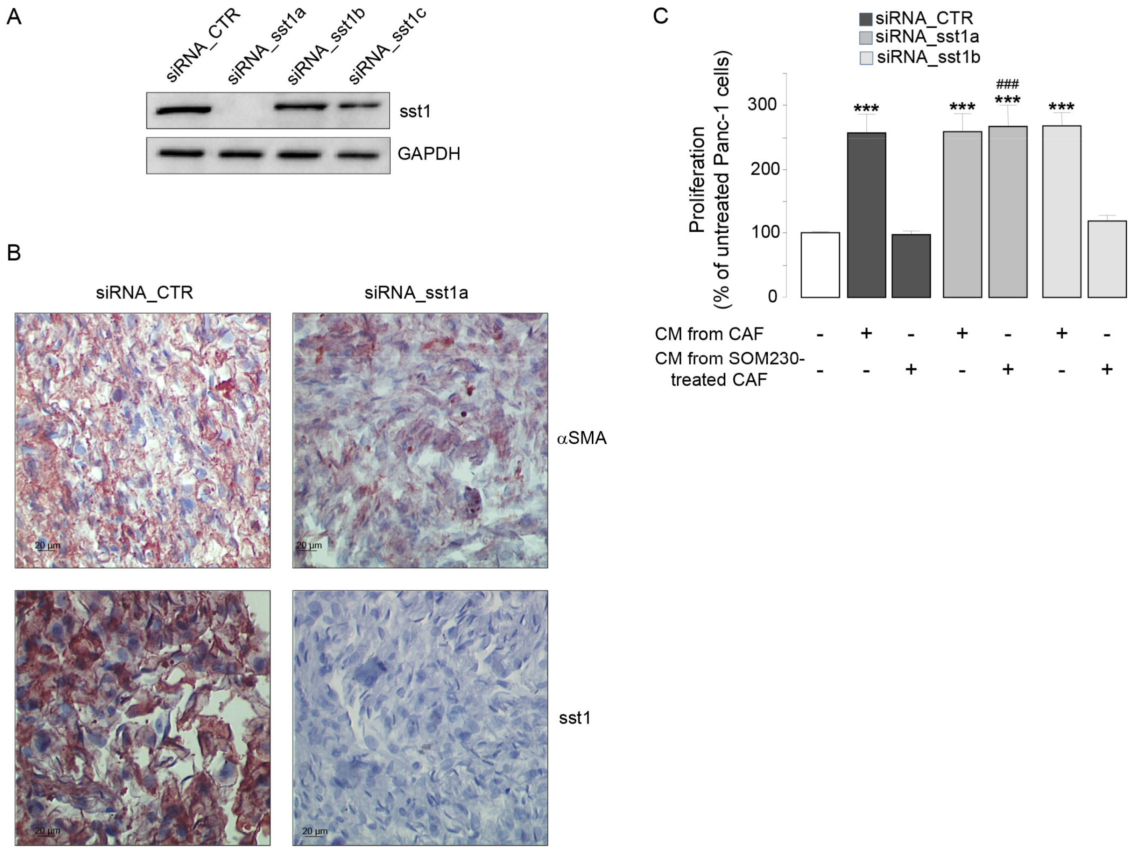

Supplementary Figure S4: A. Immunoblotting using anti-sst1 or GAPDH (loading control) antibody of protein extracts from CAFs transfected with siRNA targeting sst1 (siRNA_sst1a, or siRNA_sst1b, or siRNA_sst1c) or control (siRNA_CTR) (representative of n=3). **B.** Immunohistochemistry analysis using an anti-αSMA or -sst1 antibody in paraffin-embedded CAFs transfected with siRNA-CTR or siRNA-sst1a. **C.** Panc-1 cell viability was assessed by MTT. Cells were incubated in the presence of CM from untreated or SOM230-treated CAFs transfected with the siRNA_CTR or siRNA_sst1a or siRNA_sst1b. Results are presented as a percentage of the survival observed in the condition where cancer cells were not exposed to CAF CM (=100%) (n=3). *: effect of treatment *vs.* untreated cells; #: siRNA_CTR *vs.* siRNA_sst1.

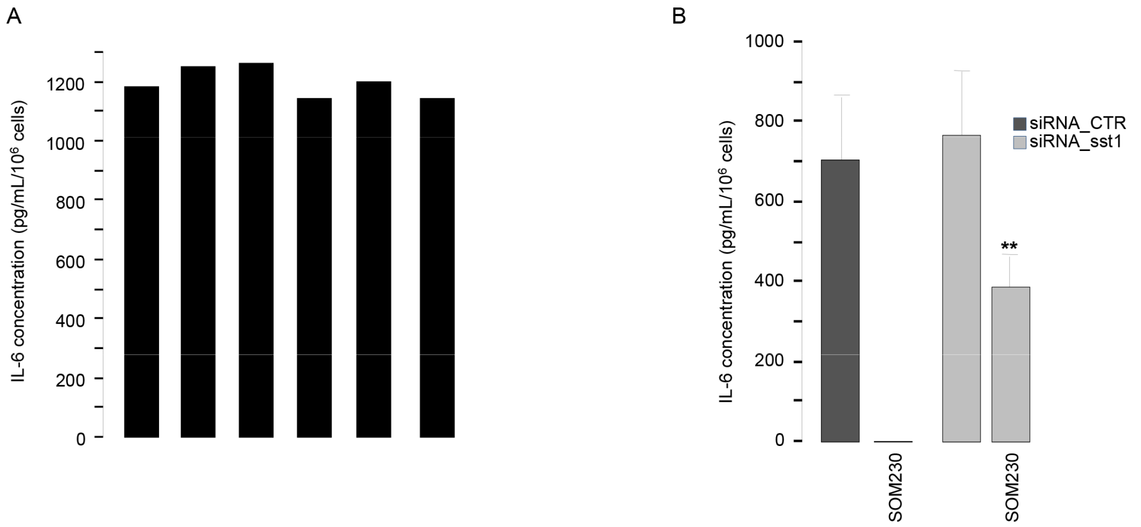

Supplementary Figure S5: A, B. ELISA assay for IL-6 in the conditioned media from six different primary cultures of CAFs **A.**, or from CAFs transfected with the siRNA_CTR or siRNA_sst1a, and treated or not with SOM230 (n=3) **B.** *: siRNA_CTR vs. siRNA_sst1.
